# The crucial role of SEMA3F in suppressing the progression of oral squamous cell carcinoma

**DOI:** 10.1186/s11658-017-0064-y

**Published:** 2017-12-28

**Authors:** Yi Liu, Ronghua Li, Kai Yin, Gang Ren, Yongdong Zhang

**Affiliations:** Department of Stomatology, Tianjin First Center Hospital, Tianjin, 300192 People’s Republic of China

**Keywords:** Oral cancer, SEMA3F, Cell proliferation, Migration, Invasion, MTT assay, Transwell assay, Xenograft model

## Abstract

**Background:**

Oral squamous cell carcinoma (OSCC) is one of the most common types of malignancy. Semaphorin 3F (SEMA3F) is highly conserved but present at a lower level in various cancers than in healthy tissues. While it has been reported that SEMA3F is involved in cancer cell proliferation, migration and invasion, its function in OSCC remains unknown.

**Methods:**

The expression of SEMA3F in OSCC tissues and OSCC-derived cells was analyzed using qRT-PCR and western blotting. Using SAS and HSC2 cells, we also monitored the effect of SEMA3F on OSCC cell proliferation, migration and invasion using MTT, colony formation and transwell assays. The function of SEMA3F in OSCC tumor formation was also assessed in vivo*.*

**Results:**

SEMA3F was significantly downregulated in OSCC tissues and OSCC-derived cells. SEMA3F shows growth inhibitory activity in SAS and HSC2 cells and may act as a tumor suppressor. It can inhibit the migration and invasion potential of OSCC cells. Our results also demonstrate that SEMA3F can suppress the growth of OSCC cells in vivo*.*

**Conclusions:**

This study revealed that SEMA3F plays a role as a tumor suppressor in OSCC cell proliferation, migration and invasion. Our finding provides new insight into the progression of OSCC. Therapeutically, SEMA3F has some potential as a target for OSCC treatment, given sufficient future research.

## Background

Oral squamous cell carcinoma (OSCC) is the most common malignant neoplasm of the oral cavity [1, 2]. According to the recent investigation of its pathogenesis and management, OSCC affects approximately 300,000 individuals per year worldwide, and the five-year survival rate for patients remains as low as 60% [3–5]. The presence of metastatic spread to the regional lymph nodes strongly correlates with a poor overall prognosis [6–8].

Despite considerable advances in the treatment of OSCC over the past two decades, the understanding of its pathogenesis is limited [9, 10]. Recent investigations of molecular alterations in various oncogenes and antitumor genes associated with OSCC development may help to address this [3, 11, 12].

Semaphorin 3F (SEMA3F), was first identified as a repulsive factor with a role in axonal guidance and neuronal development, where it modulates cell polarization and migration [13]. It has been reported that the SEMA3F gene is on chromosome 3p21.3 and that it is commonly deleted during cancer development [14–17]. SEMA3F is one of the microenvironmental factors with a tumor suppressor function [18–20].

The expression of SEMA3F in cancer cells inhibits tumor growth, invasion and metastasis [21–25]. It has been reported that SEMA3F was an inhibitor of OSCC progression [26]. However, there is very little evidence for a correlation between SEMA3F levels and OSCC progression.

In this study, we tested SEMA3F expression in 16 OSCC tissues and 4 OSCC-derived cell lines via qRT-PCR, and found that SEMA3F was downregulated in OSCC tissues and OSCC-derived cell lines. We then validated that SEMA3F suppressed OSCC cell growth, invasion and migration. Our data further demonstrated that SEMA3F inhibited OSCC tumor formation in vivo*.* These findings provide new insight into the mechanism of OSCC pathogenesis.

## Materials and methods

### Patient characteristics

Tissues were obtained from 30 OSCC patients at the Tianjin First Center Hospital. Written informed consent was obtained from all patients and the study was approved by the ethics committees of the Tianjin First Center Hospital.

### Immunohistochemistry

The OSCC tissues were embedded with paraffin and sectioned. The sections were subjected to a routine three-step immunohistochemical (IHC) staining procedure. The slides were incubated with rabbit anti-SEMA3F antibody (1:200 dilution; Sigma) and anti-Ki67 antibody (1:200 dilution; Proteintech Group) at 4 °C overnight. Then, the slides were incubated with horseradish peroxidase-labelled secondary antibody for 30 min at room temperature. The chromogen 3,3′-diaminobenzidine tetrachloride (DAB; Zhongshanjinqiao) was used as a substrate.

The cell nucleus was dyed with Harris hematoxylin solution. Levels of SEMA3F expression were determined based on the extent and intensity of staining with the scoring using this scale: negative for <5%, weak for 5–25%, mid for 25–50%, distinct for 50–75%, and strong for ≥75%. The staining intensity and stained area percentage were multiplied to produce a weighted score. Three independent evaluators assessed the scoring.

### Cell culture

We used 4 human OSCC-derived cell lines: SAS, Ca9–22, HSC2 and HSC4. One normal oral keratinocyte strain was obtained from a patient who had undergone dental surgery. This served as the control. The patient provided written informed consent prior to the start of the study. The cell lines were cultured in DMEM (Thermo-Fisher) with 10% fetal bovine serum (FBS) plus 100 IU/ml penicillin and 100 μg/ml streptomycin at 37 °C in a humidified atmosphere with 5% CO_2_.

### Plasmid construction

The CDS sequence of SEMA3F was obtained via PCR using specific primers. The PCR products were cloned into the pcDNA3.1(+) vector. The plasmid was named pcDNA-SEMA3F. The primers were: SEMA3F forward: 5’-CTAGCTAGCATGCTTGTCGCCGGTC-3′; SEMA3F reverse: 5’-CTAGTCTAGATCATGTGTCCGGAG-3′.

### Cell transfection

Transfection of cells was performed using Lipofectamine 2000 (Invitrogen) according to the manufacturer’s protocol. Briefly, cells were seeded in 6-well plates at 30–40% confluence 24 h prior to transfection. pcDNA-SEMA3F (2 μg per well) and the control were used for each transfection.

For stable transfection, HSC2 cells were selected in G418 (800 mg/ml). Individual G418-resistant colonies were isolated after 15 days of culture. Cells were treated with G418 (200 mg/ml) and maintained in culture until needed for subcutaneous inoculation into mice.

### MTT and colony formation assays

The MTT assay was performed daily over 3 days to evaluate cell proliferation. First, cells were transfected with plasmids (control or pcDNA-SEMA3F). After 24 h, cells were seeded into 96-well plates (3 × 10^3^ cells/well). Next, the cells were incubated with 25 μl of MTT (5 mg/ml, Sigma) at 37 °C for 4 h, the supernatants were removed, and 150 μl methylsulfoxide (DMSO; Sigma) was added to each well. The absorbance value (OD) of each well was measured at 490 nm.

For the colony formation assay, cells (5 × 10^5^ cells per well) were seeded in 6-well plates and transfected with pcDNA-SEMA3F (2 μg per well) or the control for 24 h. The medium was refreshed every 3 days. After 2–3 weeks of culture, the colonies were fixed with methanol, stained with 1.25% crystal violet and counted under a light microscope. All experiments were performed three times and the average results were calculated.

### Quantitative RT-PCR

Total RNA was extracted using Trizol reagent (Takara). Then, 1 μg of the RNA was converted to cDNA using Revert Acid Reverse Transcriptase (Fermentas). Real-time PCR was conducted using a Sigma-Aldrich FastStart Universal SYBR Green Master (ROX) Kit according to the manufacturer’s instructions. Double-stranded DNA specific expression was tested by the comparative Ct method using 2-ΔΔCt. The sequence of the primers used were: SEMA3F forward: 5’-CTCTGGGCTTCCCTACTGAC-3′; reverse: 5’-CACTCGCCGTTGACATCC-3′; GAPDH forward: 5’-ATCACCATCTTCCAGGAGCG A-3′; reverse: 5’-CCTTCTCCATGGTGGTGAAGAC-3′. Experiments were performed in triplicate.

### Western blot

Cellular proteins were extracted in RIPA buffer (Biomed) after transfection for 48 h. Proteins were separated by gel electrophoresis and transferred to membranes, which were then incubated with primary antibodies. The concentrations and sources of the antibodies were: rabbit polyclonal anti-NRP2 (1:2000; Abclonal), rabbit polyclonal anti-SEMA3F (1:2500; Sigma) and mouse monoclonal antihuman anti-GAPDH (1:2000; Abcam). Treatment with secondary antibodies diluted in PBST was done at room temperature for 1 h. Membranes were washed in PBST and the bound antibody was detected using an enhanced chemiluminescence system. The experiments were repeated three times.

### Migration assay

Cells were transfected with pcDNA-SEMA3F and the control. After 48 h, the cells were seeded on the upper chamber of an 8-μm pore size transwell insert (Costar) at 2.5 × 10^4^ cells/insert in DMEM. The lower chamber contained DMEM (600 μl) with 10% FBS. The migrated cells were fixed with 4% paraformaldehyde in PBS and stained in 0.5% crystal violet. The membranes were mounted on a microscope slide. Migrated cells were counted and photographed. The percentage of migrated cells was calculated. The migration index was expressed relative to the control cells. All the experiments were carried out three times and the results were expressed as the means ± SD.

### Cell invasion assay

Cell invasion assays were performed in 24-well plates using transwell chambers (8.0 μm pore size, Millipore) were coated with matrigel. The cells were transfected and then seeded in the upper chamber at a density of 2 × 10^5^ cells/ml in 400 μl of medium containing 0.5% FBS. Medium with 10% FBS was added to the lower chamber. Following 24 h incubation at 37 °C with 5% CO_2_, the invading cells were fixed in 100% methanol and stained with 0.5% crystal violet. Photographs were taken randomly for at least four fields of each membrane. The number of invading cells was expressed as the average number of cells per microscopic field over four fields.

### In vivo tumor xenograft model

A total of twelve nude mice (4-week old BALB/c nude mice; Huafukang) were randomly divided into two groups. HSC2 cells stably transfected with pcDNA-SEMA3F and control were inoculated subcutaneously into the flanks of nude mice. The length and width of tumors were taken with Vernier calipers every five days, and the mice were euthanized after thirty days. The volume of the implanted tumor was calculated using the formula: volume = (length × width^2^)/2. All animals were treated according to the guidelines established by the National Institutes of Health Guide (NIH).

### Statistical analysis

Results were analyzed statistically using Student’s *t*-test for comparisons between two groups. Data are presented as the means ± SD. Analyses were performed using SPSS 20.0. *p* < 0.05 and *p* < 0.01 were considered to be statistically significant. Unless indicated, the results shown in the figures are representative. All the experiments were done at least three times.

## Results

### Expression of SEMA3F in oral cancer tissue samples and cells

**Fig. 1:**
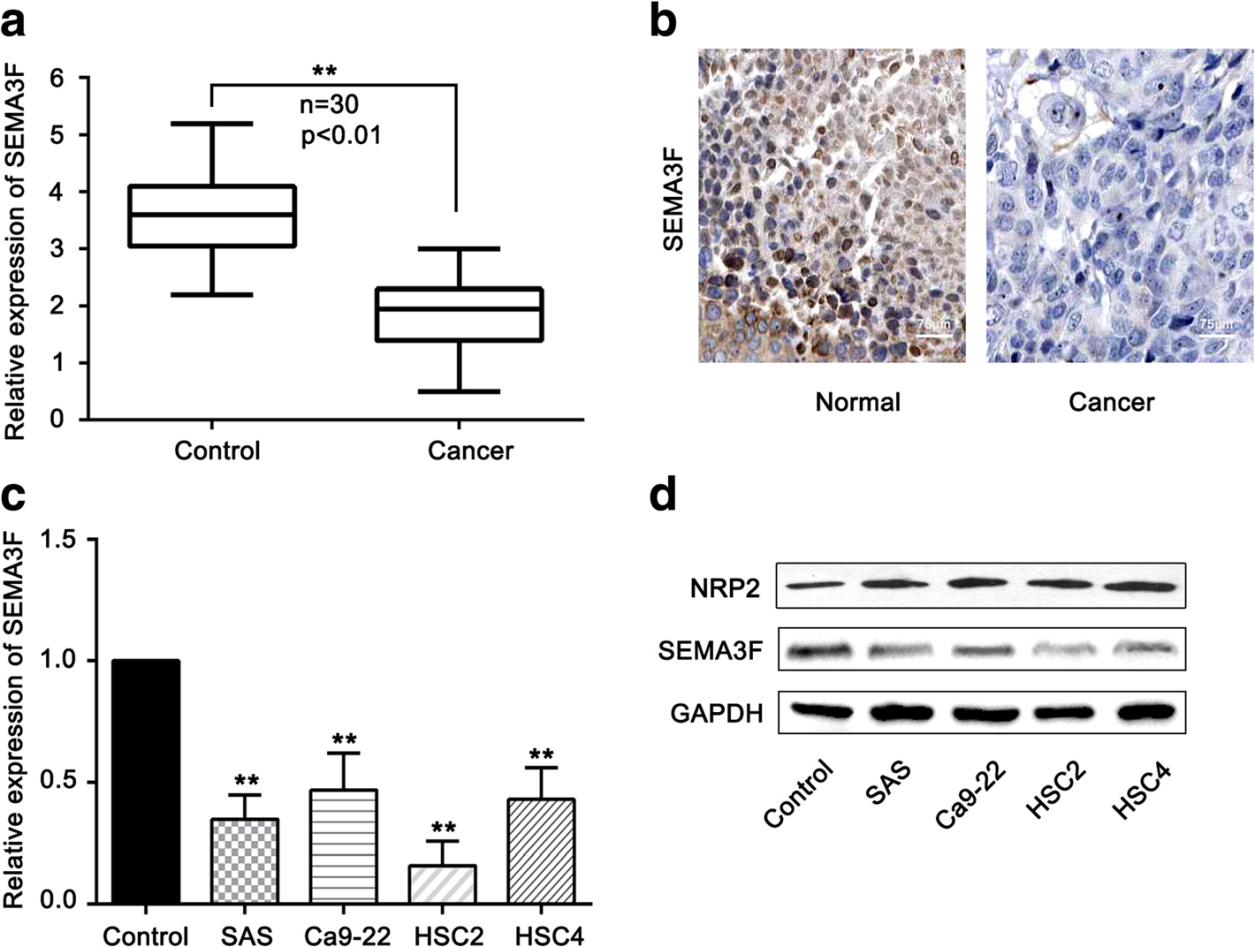
Expression of SEMA3F in oral cancer tissue samples and cells. **a** – qRT-PCR analysis of the expression of SEMA3F in 30 OSCC tissues and adjacent normal tissues. **b** – The expression of SEMA3F was tested in OSCC tissues and adjacent normal tissues using IHC. Scale bar is 75 μm. **c** – qRT-PCR analysis of the expression of SEMA3F in 4 OSCC-derived cell lines and one normal cell line. **d** – Western blotting showing the protein levels of NRP2 and SEMA3F in 4 OSCC-derived cell lines and one normal cell line. **p < 0.01 as compared with the control. The experiments were run three times

To identify the role of SEMA3F in oral carcinogenesis, we tested the expression levels of SEMA3F in a cohort of OSCC tissues and adjacent normal tissues using qRT-PCR. Our data showed that the SEMA3F mRNA levels were significantly lower in OSCC tissues than in noncancerous tissues (Fig. 1a). We also analyzed the protein level of SEMA3F in OSCC tissues and their adjacent noncancerous tissues via IHC. SEMA3F expression was generally lower in OSCC tissues than in adjacent normal tissues (Fig. 1b).

In addition, we tested the expression of SEMA3F in 4 human OSCC-derived cell lines (SAS, Ca9–22, HSC2, and HSC4) and one normal oral keratinocyte strains (control) using qRT-PCR (Fig. 1c). The expression of SEMA3F in the 4 OSCC-derived cell lines was tested using western blot technology (Fig. 1d). We also tested the expression level of NRP2, a receptor of SEMA3F, in the above-mentioned OSCC cells. We found that NRP2 was significantly upregulated in OSCC cells compared with control cells (Fig. 1d).

These results show that SEMA3F was significantly downregulated in OSCC cells compared with control cells at both the mRNA and protein levels. This suggests that SEMA3F may be a tumor suppressor in OSCC.

### SEMA3F inhibits cell proliferation in oral cancer cells

**Fig. 2:**
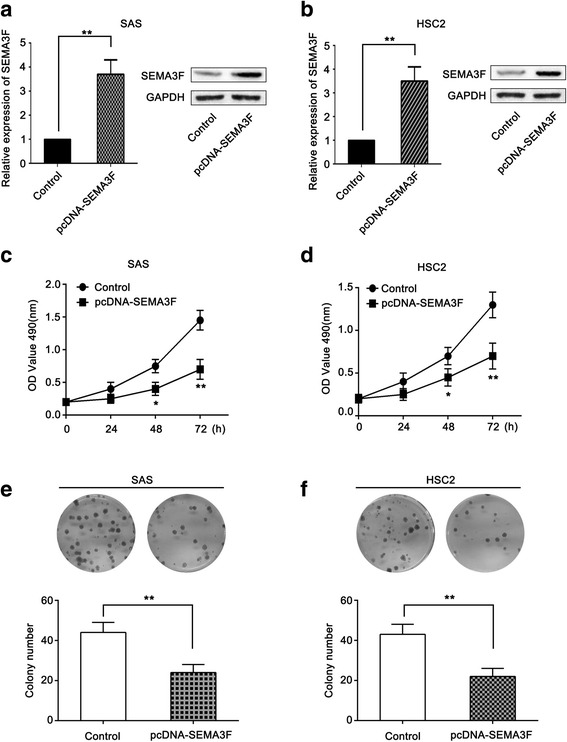
SEMA3F inhibits cell proliferation in oral cancer cells. **a** – qRT-PCR and western blotting analysis of the transfection efficiency of pcDNA-SEMA3F in SAS cells. **b** – qRT-PCR and western blotting analysis of the transfection efficiency of pcDNA-SEMA3F in HSC2 cells. **c** – MTT assays of cell viability in SAS cells transfected with pcDNA-SEMA3F and the control. **d** – MTT assays of cell viability in HSC2 cells transfected with pcDNA-SEMA3F and the control **e** – Colony formation analysis of SAS cells transfected with pcDNA-SEMA3F and the control. **f** – Colony formation analysis of HSC2 cells transfected with pcDNA-SEMA3F and the control. *p < 0.05; **p < 0.01 as compared with the control. The experiment was run three times

To detect the effect of SEMA3F on OSCC cell growth, we cloned the SEMA3F CDS and transfected it into the human OSCC cell lines SAS and HSC2. Increased expression of SEMA3F upon transfection was confirmed using qRT-PCR and western blot (Fig. 2a and b). As demonstrated in MTT assays, SEMA3F restoration dramatically inhibited OSCC cell proliferation (Fig. 2c and d). The inhibitory effect of SEMA3F on OSCC cell growth was further confirmed with colony formation assays. The number of colonies for cells transfected with pcDNA-SEMA3F was significantly lower than for cells transfected with the control (Fig. 2e and f). This means that SEMA3F inhibits the growth ability in SAS and HSC2 cells and acts as a potential OSCC tumor suppressor.

### SEMA3F suppresses oral cancer cell migration and invasion

**Fig. 3:**
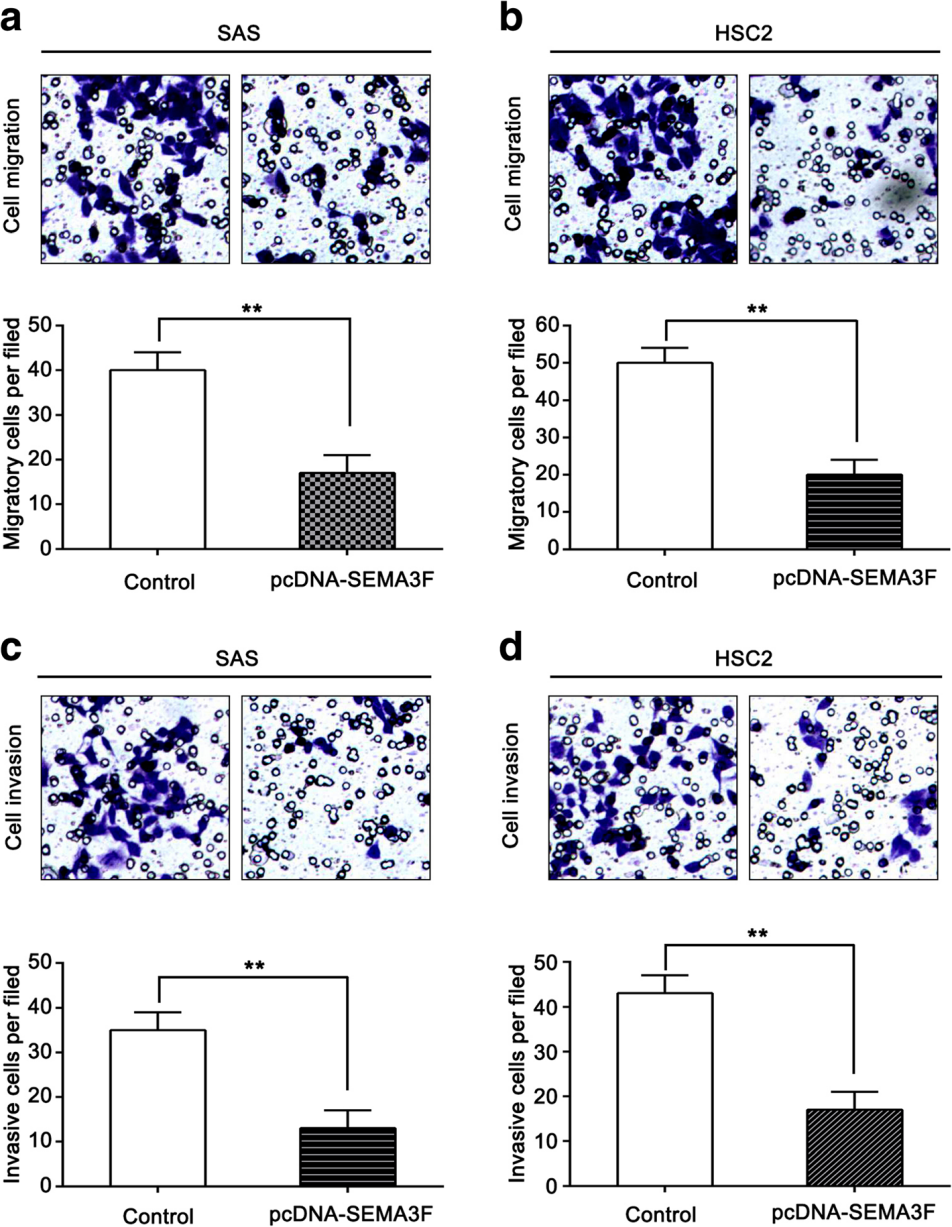
SEMA3F suppresses oral cancer cell migration and invasion. **a** – Transwell assays of cell migration in SAS cells. **b** – Transwell assays of cell migration in HSC2 cells. **c** – Transwell assays of cell invasion in SAS cells. **d** – Transwell assays of cell invasion in HSC2 cells. **p < 0.01 as compared with the control. The experiment was run three times

We attempted to identify the effect of SEMA3F on OSCC cell migration and invasion according to the potent capacity of OSCC to invade locally and form distant metastases. We used the transwell migration assay for this experiment. Our results showed that the rate of migration for SAS and HSC2 cells transfected with pcDNA-SEMA3F was significantly lower than for control transfected cells (Fig. 3a and b). Furthermore, the transwell invasion assays showed that the number of cells that passed through Matrigel-coated membrane into the lower chamber was significantly lower for cells transfected with pcDNA-SEMA3F than for the control transfected cells (Fig. 3c and d). These results suggest that SEMA3F can inhibit the migration and invasion potential of OSCC cells.

### Effect of SEMA3F expression on the progression of oral cancer in vivo

**Fig. 4:**
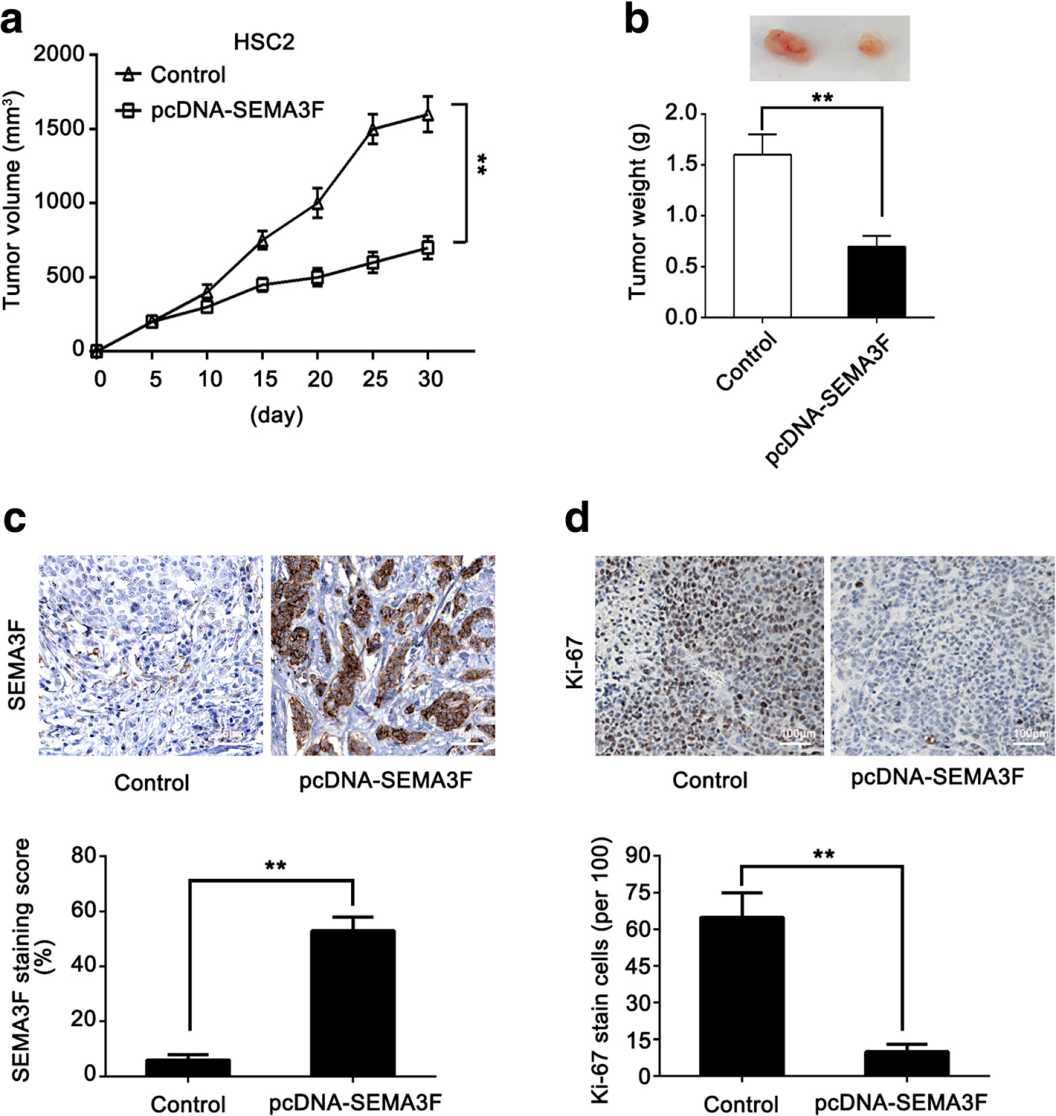
Effect of SEMA3F expression on the progression of oral cancer in vivo. **a** – HSC2 cells transfected with pcDNA-SEMA3F were transplanted into nude mice. The growth curve and average weight of tumors are shown. **b** – HSC2 cells transfected with the control were transplanted into the nude mice. The growth curve and average weight of tumors are shown. **c** – The expression of SEMA3F was tested using IHC analysis in tumor tissues from the nude mice. The scale bar is 75 μm. **d** – The expression of Ki67 was tested using IHC analysis in tumor tissues from the nude mice. The scale bar is 100 μm. **p < 0.01 as compared with the control

To explore whether changes in SEMA3F expression could influence the growth of OSCC tumors in vivo, two groups of nude mice were subcutaneously injected with HSC2 cells stably transfected with pcDNA-SEMA3F and control plasmids. Tumor formation and weight were assessed in these two groups. Our results showed that the ectopic expression of SEMA3F inhibited OSCC tumorigenesis in vivo (Fig. 4a). The average tumor weight of mice inoculated with pcDNA-SEMA3F-transfected HSC2 cells was significantly lower than that of mice inoculated with the control cells (Fig. 4b).

We also tested the expression levels of SEMA3F in the tumors from mice using the IHC assay. Our data revealed that SEMA3F levels were higher in pcDNA-SEMA3F-transfected HSC2 cells group than in the control group (Fig. 4c). In addition, we tested the expression levels of Ki-67, a biomarker of cell growth, in the tumor tissues. Our results show that the Ki-67 expression level was significantly lower in the pcDNA-SEMA3F-transfected HSC2 cells group than in the control group (Fig. 4d). Thus, we conclude that SEMA3F is able to suppress the growth of OSCC cells in vivo.

## Discussion

Recent study of SEMA3F has shown that it can play crucial roles in the regulation of cell proliferation, migration and invasion [21, 22, 27, 28]. SEMA3F is causally downregulated in the initiation and progression of some cancers [20, 22, 23, 27]. However, the role of SEMA3F in oral carcinogenesis remains unkown. In this study, we attempted to investigate the expression of SEMA3F in OSCC tissues and adjacent normal tissues, and explored its biological function in oral carcinogenesis.

Firstly, we tested the expression of SEMA3F in 30 pair of OSCC tissues and adjacent normal tissues. Our data show that SEMA3F is significantly downregulated in OSCC tissues compared with adjacent normal tissues. The relative expression of SEMA3F in 4 human OSCC-derived cell lines (SAS, Ca9–22, HSC2 and HSC4) was also significantly downregulated compared with normal control cells.

Zhang et al. demonstrated that the expression level of SEMA3F is downregulated in OSCC cancer tissues [26]. Our results are consistent with that and other earlier reports. This suggests that SEMA3F is a candidate tumor suppressor in the pathogenesis of OSCC.

It has been reported that NRP2 is a receptor of SEMA3F, so we tested the expression levels of NRP2 and SEMA3F in OSCC cells. We found that the NRP2 protein level was upregulated but that the SEMA3F protein level was downregulated. This suggests that there would be a SEMA3F/NRP2 axis pathway during the progression of OSCC.

Functional studies were required to clarify the role of SEMA3F in the development of OSCC cells. We tested the function of SEMA3F, as a putative tumor suppressor, in SAS and HSC2 cell lines. Overexpression of SEMA3F in SAS and HSC2 cells shows a significant growth-suppressing effect through the inhibition of cell proliferation and colony formation.

It has been reported that SEMA3F suppressed the migration and invasion of several cancers [29–32]. To better understand the tumor suppressive effect of SEMA3F in oral tumorigenesis, we identified the function of SEMA3F in SAS and HSC2 cell lines using transwell analysis. Notably, we found that SEMA3F inhibits OSCC cell migration and invasion. These data suggest that SEMA3F inhibits growth, migration and invasion in SAS and HSC2 cells and acts as a potential tumor suppressor in OSCC pathogenesis.

In addition, we observed that overexpression of SEMA3F could significantly decrease the OSCC tumor volume and weight in vivo*.* Our data confirmed that Ki-67, a marker of proliferation, was induced in the restoration of the SEMA3F group. Thus, we conclude that SEMA3F is able to suppress the proliferation of OSCC in vivo.

In summary, our data show that SEMA3F is significantly downregulated in OSCC tissues and OSCC-derived cells. Our study is the first to demonstrate that SEMA3F inhibits OSCC cell growth, migration and invasion in vitro and in vivo. This means that SEMA3F may be a potential biomarker for OSCC diagnosis and could serve as a new target for OSCC therapy in the future.

## Conclusion

We show that the SEMA3F plays an important role in OSCC cell proliferation, migration and invasion. Our finding is definitely important for studies on the development of OSCC, since it may provide new insight into the role of SEMA3F in OSCC cancer progression and be helpful in the development of further targeted drugs for OSCC cancer treatment.

## Abbreviations

FBS: Fetal bovine serum
GAPDH: Glyceraldehyde-3-phosphate dehydrogenase
MTT: 3-(4,5-dimethyl-2-thiazolyl)-2,5-diphenyl-2-H-tetrazolium bromide
OD: Absorbance value
OSCC: Oral squamous cell carcinoma
qRT-PCR: quantitative reverse transcription PCR
SEMA3F: Semaphorin 3F
